# Androgens augment pulmonary responses to ozone in mice

**DOI:** 10.14814/phy2.14214

**Published:** 2019-09-22

**Authors:** Ross S. Osgood, David I. Kasahara, Hiroki Tashiro, Youngji Cho, Stephanie A. Shore

**Affiliations:** ^1^ Department of Environmental Health Harvard T.H. Chan School of Public Health Boston Massachusetts

**Keywords:** Airway hyperresponsiveness, castration, flutamide, IL‐1α

## Abstract

Ozone causes airway hyperresponsiveness, a defining feature of asthma, and is an asthma trigger. In mice, ozone‐induced airway hyperresponsiveness is greater in males than in females, suggesting a role for sex hormones in the response to ozone. To examine the role of androgens in these sex differences, we castrated 4‐week‐old mice. Controls underwent sham surgery. At 8 weeks of age, mice were exposed to ozone (2ppm, 3 h) or room air. Twenty‐four hours later, mice were anesthetized and measurements of airway responsiveness to inhaled aerosolized methacholine were made. Mice were then euthanized and bronchoalveolar lavage was performed. Castration attenuated ozone‐induced airway hyperresponsiveness and reduced bronchoalveolar lavage cells. In intact males, flutamide, an androgen receptor inhibitor, had similar effects to castration. Bronchoalveolar lavage concentrations of several cytokines were reduced by either castration or flutamide treatment, but only IL‐1α was reduced by both castration and flutamide. Furthermore, an anti‐IL‐1α antibody reduced bronchoalveolar lavage neutrophils in intact males, although it did not alter ozone‐induced airway hyperresponsiveness. Our data indicate that androgens augment pulmonary responses to ozone and that IL‐1α may contribute to the effects of androgens on ozone‐induced cellular inflammation but not airway hyperresponsiveness.

## Introduction

Ozone (O_3_) is an asthma trigger. Exposure to O_3_ induces asthma symptoms and high ambient O_3_ concentrations are associated with increased emergency room visits and hospital admissions for asthma (Holtzman et al., 1979; Fauroux et al., 2000; Alexis et al., 2000; Bell et al., 2005). In both humans and mice, O_3_ causes damage to the airway epithelium, release of acute phase cytokines such as IL‐1α, recruitment of neutrophils and macrophages into the lungs, and airway hyperresponsiveness (AHR), a cardinal feature of asthma (Holtzman et al., 1979; Devlin et al., 1991; Kasahara et al., 2015a; Michaudel et al., 2018).

There is evidence for sex differences in the pulmonary response to O_3_. For example, the association between long‐term O_3_ exposure and reductions in lung function is stronger in young adult males than females (Galizia and Kinney, 1999). The association between ambient O_3_ exposure and emergency room visits for asthma also exhibits both sex and age dependency (Paulu and Smith, 2008; Sheffield et al., 2015). For example, Sheffield (Sheffield et al., 2015) reported an association between O_3_ and emergency visits for boys across all ages, whereas in girls the association was only evident after the age of 10. Sex differences in the response to O_3_ have also been reported in mice. Following O_3_ exposure, females have higher pulmonary expression of the inflammatory cytokines MIP‐1α and IL‐6, than males (Cabello et al., 2015; Mishra et al., 2016). In females, hormonal status can also alter the lung microRNA profiles of O_3_‐exposed mice (Fuentes et al., 2018). There are also sex differences in O_3_‐induced airway hyperresponsiveness. In C57BL/6 mice, the magnitude of O_3_‐induced AHR is greater in male than female mice, even though there is no effect of sex on indices of O_3_‐induced lung injury (Cho et al., 2018a; Birukova et al., 2019). In female mice, the stage of the estrous cycle influences O_3_‐induced AHR: compared to mice in the luteal stage, mice in the follicular stage of the estrous cycle, when estradiol and luteinizing hormone are elevated, have increased O_3_‐induced AHR (Fuentes et al., 2019). In contrast, progesterone, which is elevated in the luteal stage of the estrous cycle, does not associate with induced AHR (Birukova et al., 2019). These data indicate a role for female sex hormones in the response to O_3_. In contrast, the impact of androgens on O_3_‐induced AHR has not been evaluated.

The purpose of this study was to examine the role of androgens in the pulmonary response to O_3_. To do so, we manipulated sex hormones in male mice by castration or treatment with the androgen receptor inhibitor, flutamide. Our data indicated that both castration and flutamide reduced O_3_‐induced AHR and inflammatory cell recruitment, suggesting a role for androgens in these events. BAL concentrations of several cytokines were affected by either castration or flutamide but only IL‐1α was affected by both castration and flutamide. For this reason, and because IL‐1α has been previously linked to O_3_‐induced AHR in female mice (Michaudel et al., 2018), we treated male mice with an IL‐1α neutralizing antibody 24 h prior to O_3_ exposure. Our data indicated that anti‐IL‐1α did attenuate O_3_‐induced neutrophil recruitment to the lungs, but did not alter O_3_‐induced AHR.

## Methods

### Animals

This study was approved by the Harvard Medical Area Standing Committee on Animals. Male C57BL/6J mice were purchased from The Jackson Laboratories (Bar Harbor, ME) where removal of the gonads by castration was performed at 4 weeks of age. Controls underwent sham surgery. No antibiotics were used during recovery. Mice were transferred to our vivarium at 5 weeks of age, and housed there until exposure at 8 weeks of age. For experiments involving flutamide and anti‐IL‐1α treatment, intact male C57BL/6 mice from Jackson Labs were used. All mice were housed under a 12/12 h light/dark cycle and fed mouse chow (Picolab Rodent diet 5053) and housed with at least two mice per cage.

### Protocol

Three protocols were used. In the first, castrated and sham‐castrated male mice were exposed to room air or O_3_ (2 ppm for 3 h). O_3_ exposure was begun between 9 and 10 o'clock AM. Twenty‐four hours later, mice were anesthetized for the measurement of airway responsiveness to inhaled aerosolized methacholine. This time point was chosen because O_3_‐induced AHR, our primary endpoint of interest, is consistently observed in males at this time (Birukova et al., 2019). Following measurement of airway responsiveness, mice were euthanized with an overdose of sodium pentobarbital, blood was harvested by cardiac puncture for the preparation of serum, and BAL was performed. The lungs were then harvested. One lung was immersed in RNAlater (Qiagen, Hilden, Germany) and stored at −80°C until used to isolate RNA. The other was frozen in liquid nitrogen until used to assess protein carbonyls.

In the second protocol, gonadally intact 8‐week‐old male mice were implanted subcutaneously using a trocar with a pellet containing the androgen receptor inhibitor, flutamide (5 mg pellet), or a placebo control pellet (Innovative Research of America, Sarasota, FL). This system provides continuous release of the substances in the pellets for up to 3 weeks. Two weeks after implantation, mice were exposed to O_3_ and evaluated. The dose of flutamide was chosen based on its efficacy in other androgen‐dependent models (Zhu et al., 2010). To confirm the effectiveness of the flutamide treatment in the lungs, we examined pulmonary mRNA abundance of an androgen receptor dependent gene, *Fkpb5*.

In the third protocol, male C57BL/6 mice, aged 10–12 weeks, were treated with an IL‐1α neutralizing antibody (anti‐mouse IL‐1α, 200 µg per mouse delivered i.p.; BioXCell, West Lebanon, NH, clone ALF‐161) or an equal amount of isotype antibody (polyclonal Armenian hamster IgG; BioXCell Cat# BE0091). This antibody dosing regimen decreases O_3_‐induced AHR and BAL neutrophils in female mice (Michaudel et al., 2018), but its effect in males has not been examined. Twenty‐four hours later, mice were exposed to O_3_ and evaluated.

Experiments were replicated with two cohorts of ozone‐exposed sham and castrated mice, four cohorts of ozone‐exposed placebo and flutamide‐treated mice, and seven cohorts of anti‐IL‐1α‐treated ozone‐exposed mice.

### Ozone exposure

During exposure, mice were placed in individual wire mesh cages within a stainless steel and Plexiglas chamber and exposed to O_3_ (2ppm, 3h) or room air. Food and water were removed during exposure. Following exposure, mice were immediately returned to clean cages and food and water were restored (Lu et al., 2006). This O_3_ concentration was chosen because it results in consistent O_3_‐induced AHR, whereas lower concentrations do not (Birukova et al., 2019). In mice, an O_3_ exposure of 2 ppm for 3 h produces an inhaled O_3_ dose that is equivalent to that used in human chamber studies evaluating effects of O_3_ on lung function in which humans are exposed to 0.4 ppm for 2 h with intermittent exercise (Hatch et al., 1994; Watkinson et al., 1996).

### Measurement of pulmonary mechanics and airway responsiveness

Twenty hours after exposure mice were anesthetized with pentobarbital and xylazine, intubated with a tubing adaptor, and ventilated (Flexivent, SCIREQ, Montreal, Canada) as previously described (Cho et al., 2018a; Cho et al., 2018b). Three volume excursions to total lung capacity (30 cm H_2_O trans‐respiratory system pressure) were administered to establish a common volume history. Then, 1 min after an excursion to total lung capacity, 10 breaths from an aerosol generated from PBS were administered. Total respiratory system resistance (*R*_RS_) and elastance (*E*) was measured every 15 s for 3 min by the forced oscillation technique (Hantos et al., 1992), as we have described (Williams et al., 2013). Following each measurement of R_RS_, measurements of total lung impedance (*Z*_L_) were obtained using a 3‐s optimized pseudorandom signal containing frequencies ranging from 0.25 to 19.63 Hz. A parameter estimation model was used to partition *Z*_L_ into components representing Newtonian resistance (Rn), which largely reflects the conducting airways, and the coefficients of respiratory system tissue damping (*G*) and respiratory system tissue elastance (*H*), which largely reflect changes in the lung tissue and peripheral airways. This sequence was repeated with aerosolized methacholine chloride in concentrations increasing from 1 to 100 mg/mL. The three highest values of *R*_RS,_ Rn, *G*, *H*, and *E* at each dose were averaged to construct dose–response curves to methacholine for each index.

### Blood collection

Blood was collected from the right ventricle via cardiac puncture with a 12‐gauge needle. Serum was prepared and stored at −80°C until analysis of corticosterone by ELISA (R&D Systems, Minneapolis, MN) and serum cytokines and chemokines by multiplex assay (Mouse Cytokine Array/Chemokine Array 31‐Plex (MD31); Eve Technologies, Calgary, AB, Canada).

### Bronchoalveolar lavage (BAL) and tissue collection

After the mice were euthanized, the lungs were lavaged with two 1‐mL aliquots of PBS as we have described (Brand et al., 2016). The lavageates were pooled and centrifuged to separate supernatant and cells. Total BAL cell counts were counted with a hemocytometer and cell differentials were determined by centrifuging BAL cells onto glass slides and staining with a Hema3 Stain kit. At least 300 cells were counted. The BAL supernatant was frozen at −80°C until analyzed for protein content by the Bradford assay (Bio‐Rad, Hercules, CA). BAL cytokines and chemokines were first concentrated approximately 8‐fold (Amicon Ultra – 0.5 mL centrifugal filters; Millipore, Tullagreen, Cork, Ireland) and then were assayed by multiplex assay (Eve Technologies).

### RT‐PCR

Lung RNA was purified using a commercial kit (RNeasy Mini Kit; Qiagen) and converted into cDNA (SuperScript III; Invitrogen, Carlsbad, CA). Lung gene expression was assessed using RT‐PCR (7300 Real‐Time PCR Systems; Applied Biosystems, Foster City, CA) with SYBR‐green detection and normalized to 36B4 ribosomal RNA, forward primer GCTCCAAGCAGA TGCAGCA, reverse primer, CCGGATGTGAGGCAGCAG (Mathews et al., 2017) using the ΔΔ Ct method. The primers for FK506‐binding protein 51 are as follows: forward primer ACTGTGTACTTCAAGGGAGGC, reverse primer, TTCTCTGACAGGCCGTATTCC. The primers for Glutathione *S*‐Transferase Alpha 1 (*Gsta1*) were as follows: forward primer ACCTGATGCACTCCATTCTG, reverse primer, GCTGGACTGTGAGCTGAGTG.

### Enzyme‐linked immunosorbent assay

We evaluated lung tissue protein carbonyls by ELISA (Oxiselect Protein Carbonyl ELISA kit, Cat# STA‐310; Cell Biolabs, Inc, San Diego, CA). First, we homogenized lung tissue in PBS and determined the concentration of protein by the Bradford assay (Bio‐Rad, Hercules, CA). We then used 1 µg of protein per sample to determine protein carbonyl levels as per kit instructions.

### Statistical analysis

The significance of differences between groups was assessed using factorial ANOVA combined with LSD Fisher post hoc analysis (Statistica Software, Tulsa, OK) using gonadal status and exposure as main effects. In cases where only O_3_ exposed animals (flutamide, anti‐IL‐1α) were evaluated, t‐tests were used to compare treated and control groups. A *P*‐value <0.05 (two‐tailed) was considered significant. Values are expressed as mean ± SE.

## Results

### Androgens augment O_3_‐induced airway hyperresponsiveness and pulmonary inflammation

**Figure 1:**
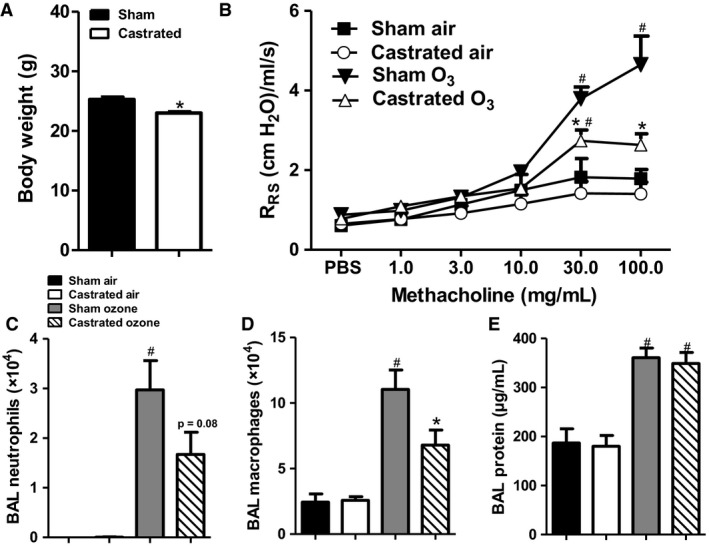
Androgens augment O_3_‐induced airway hyperresponsiveness and pulmonary inflammation. Male mice that had undergone either sham surgery or castration were exposed to room air or O_3_ (2ppm, 3h) and evaluated 24 h later. Shown are: (A) body weight prior to exposure to room air or O_3_; (B) airway responsiveness of sham or castrated mice exposed to air or O_3_; (C) BAL neutrophils; (D) BAL macrophages; (E) BAL protein. Results are mean ± SE of data from 4 air‐ and 8 O_3_‐exposed mice per group. *P < 0.05 compared with sham‐treated mice with same exposure; ^#^ *P* < 0.05 compared to air‐exposed with same type of surgery. R_RS_: respiratory system resistance.

**Figure 2:**
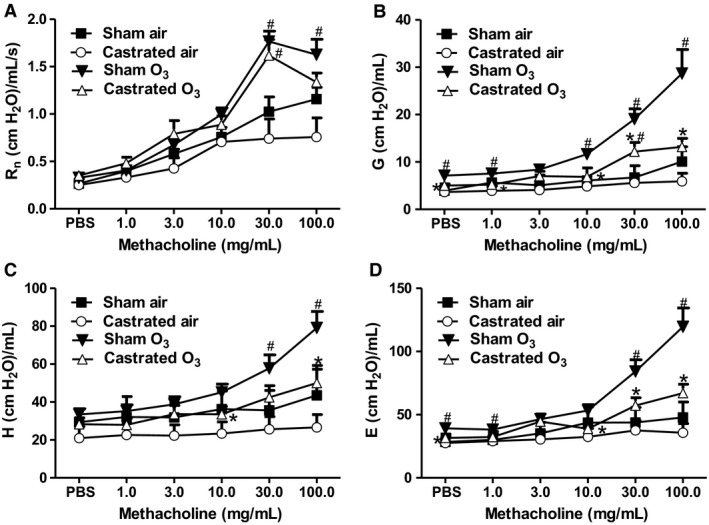
Effects of castration on O_3_‐induced AHR are mediated in the lung periphery. Male mice that had undergone either sham surgery or castration were exposed to room air or O_3_ (2ppm, 3h) and evaluated 24 h later. Shown are changes in airway responsiveness assessed using (A) Rn, (B) G, (C) H, or (D) E. Results are mean ± SE of data from 4 air‐ and 8 O_3_‐exposed mice per group. *P < 0.05 compared with sham treated mice with same exposure; ^#^ *P* < 0.05 compared to air‐exposed with same type of surgery. R_n_: Newtonian resistance; G: coefficient of respiratory system tissue damping; H: coefficient of respiratory system elastance, and E: respiratory system elastance.

Compared to sham castrated mice, body weight was significantly lower in castrated mice (Fig. 1A), consistent with the growth‐promoting effects of androgens. In air‐exposed mice, there was no significant difference in airway responsiveness assessed between castrated and sham‐castrated male mice using R_RS_ as the index of response (Fig. 1B). Compared to air, O_3_ increased airway responsiveness in both groups of mice. However, the magnitude of O_3_‐induced AHR was lower in castrated than sham‐castrated mice (Fig. 1B). Similar results were obtained using G, H, and E but not Rn as the index of response (Fig. 2), indicating that the effect of castration was on responses in the lung periphery rather than the central airways. O_3_ exposure increased BAL neutrophils and macrophages (Fig. 1C,D) and also increased BAL protein (Fig. 1E), an indicator of O_3_‐induced injury to the alveolar/capillary barrier. Compared to sham‐castrated mice, BAL macrophages were significantly lower in castrated than sham‐castrated mice exposed to O_3_ (Fig. 1D). A similar trend was observed for BAL neutrophils, but did not reach statistical significance (*P* < 0.08). There was no effect of castration on BAL protein (Fig. 1E).

**Figure 3:**
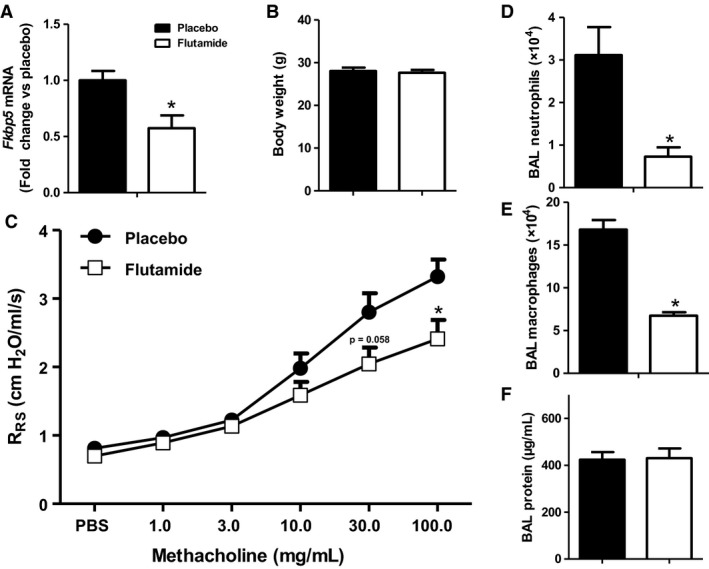
Androgen receptor inhibition attenuates O_3_‐induced airway hyperresponsiveness and pulmonary inflammation. Gonadal intact male mice were treated with placebo or 5mg of flutamide for 2 weeks prior to O_3_ exposure. Mice were evaluated 24 h after O_3_ exposure. Shown are: (A) lung mRNA abundance of expression of FK506 binding protein 51 (*Fkbp5*); (B) body weight of mice after 2 weeks of treatment with placebo or flutamide; (C) airway responsiveness of placebo or flutamide‐treated mice exposed to air or O_3_; (D) BAL neutrophils; (E) BAL macrophages; (F) BAL protein. Results are mean ± SE of data from 7–8 mice per treatment group. *P < 0.05 placebo versus flutamide treatment.

**Figure 4:**
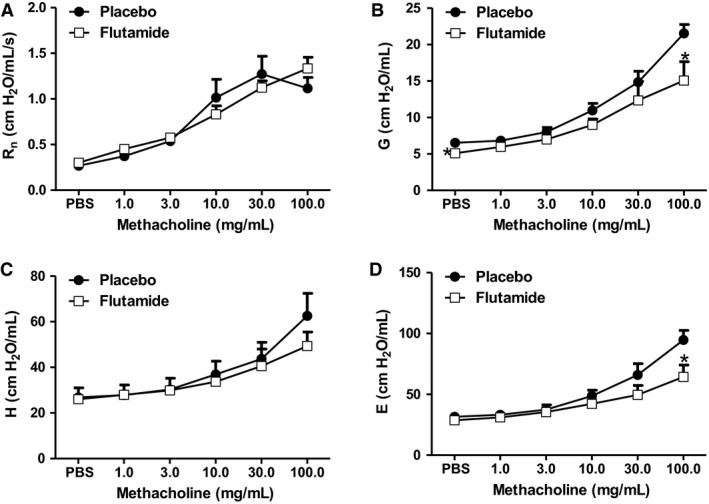
Effects of flutamide O_3_‐induced AHR are mediated in the lung periphery. Male mice treated with flutamide or placebo control were exposed to O_3_ (2ppm, 3h) and evaluated 24 h later. Shown are changes in airway responsiveness assessed using (A) Rn, (B) G, (C) H, or (D) E. Results are mean ± SE of data from 4 air‐ and 7 O_3_‐exposed mice per group. *P < 0.05 compared with sham treated mice with same exposure. R_n_: Newtonian resistance; G: coefficient of respiratory system tissue damping; H: coefficient of respiratory system elastance, and E: respiratory system elastance.

Because castration was performed at puberty, it is conceivable that castration‐induced changes in the response to O_3_ (Fig. 1) were the result of altered somatic growth and development due to loss of the anabolic effects of testosterone in the weeks between castration and evaluation, as indicated by the changes in body weight (Fig. 1A). To address this possibility, we treated gonadally intact males, with an androgen receptor inhibitor, flutamide, or placebo control, for 2 weeks prior to O_3_ exposure, but beginning after most somatic growth, including lung growth, is complete (at 8 weeks of age). Because there was no effect of castration in air‐exposed mice, we only evaluated O_3_‐exposed mice. To confirm the efficacy of the flutamide treatment, we examined pulmonary expression of FK506 binding protein 51 (*Fkpb5*), an androgen receptor dependent gene that is expressed in the lung (Magee et al., 2006; Wang et al., 2007). Compared to placebo control, flutamide caused a significant reduction in pulmonary *Fkpb5* mRNA abundance (Fig. 3A). Compared to control treatment, flutamide had no effect on body mass (Fig. 3B). Compared to placebo controls, O_3_‐induced AHR was significantly reduced in mice treated with flutamide (Fig. 3C), and as with castration, the effect occurred in the lung periphery rather than the central airways (Fig. 4). BAL neutrophils and macrophages, but not BAL protein, were also lower in flutamide‐treated than control mice (Figs. 3D‐F), similar to castrated versus sham‐castrated mice (Fig. 1C‐E). The data support the hypothesis that androgens augment pulmonary responses to O_3_, and indicate that the effect of androgens is likely not the result of effects on somatic growth and development.

### BAL IL‐1α is reduced in both castrated and flutamide‐treated mice

**Figure 5:**
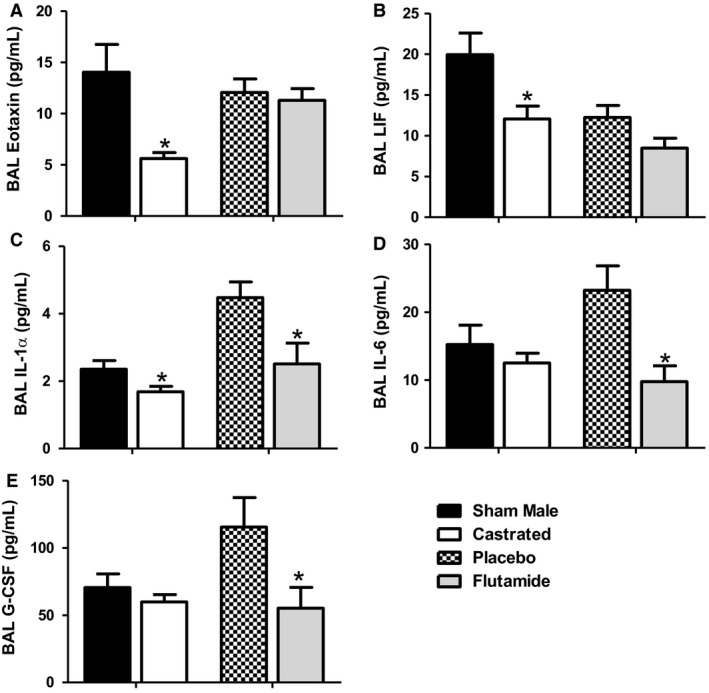
Androgens augment O_3_‐induced BAL cytokines and chemokines. Results of a multiplex assay on BAL fluid from O_3_ exposed mice that underwent sham surgery or castration as well as from placebo‐ or flutamide‐treated mice. Shown are cytokines and chemokines for which there was a significant effect either of castration or flutamide: (A) BAL Eotaxin; (B) BAL LIF; (C) BAL IL‐1α; (D) BAL IL‐6; (E) BAL G‐CSF. Results are mean ± SE of data from 6–8 mice per treatment group. *P < 0.05 compared with group control (sham or placebo).

Exposure to O_3_ induces the expression of a variety of cytokines and chemokines within the lungs and many of these cytokines and chemokines have to been shown to contribute to the ability of O_3_ to induce AHR (Shore et al., 2001; Johnston et al., 2005a; Johnston et al., 2005b; Pichavant et al., 2008; Barreno et al., 2013; Michaudel et al., 2018). To determine whether androgens augment O_3_‐induced AHR by increasing the expression of any of these inflammatory mediators, we ran a multiplex assay for 31 cytokines and chemokines on BAL fluid from castrated versus sham‐castrated mice and from flutamide‐treated versus control mice exposed to O_3_. BAL concentrations of eotaxin, leukemia inhibitory factor (LIF), and IL‐1α were each significantly lower in castrated than sham castrated mice exposed to O_3_ (Fig. 5A‐C). BAL concentrations of IL‐6, granulocyte colony‐stimulating factor (G‐CSF), and IL‐1α were each significantly lower in flutamide versus control mice exposed to O_3_ (Fig. 5C‐E). Other cytokines and chemokines known to be induced by O_3_ are shown in Table 1 and were not significantly different in O_3_‐exposed sham versus castrated or placebo‐ versus flutamide‐treated mice.

**Table 1:** BAL cytokines and chemokines in sham surgery, castrated, placebo, or flutamide‐treated O_3_‐exposed mice

pg/mL	Sham	Cast	Placebo	Flutamide	*P*‐value sham/castrated	*P*‐value placebo/flutamide
IL‐1β	1.1 ± 0.2	0.6 ± 0.1	0.1 ± 0.06	0.21 ± 0.02	0.06	0.56
IL‐5	7.7 ± 1.1	9.3 ± 0.9	4.4 ± 0.8	4.95 ± 0.7	0.26	0.66
IL‐9	1.6 ± 0.0	1.3 ± 0.1	2.0 ± 0.4	1.30 ± 0.3	0.07	0.18
CCL2	12.7 ± 1.9	8.6 ± 1.2	10.0 ± 1.6	7.10 ± 1.4	0.09	0.21
CCL3	6.1 ± 0.9	5.5 ± 1.0	6.6 ± 0.6	4.39 ± 0.9	0.64	0.09
CCL4	3.1 ± 0.4	3.9 ± 0.7	1.8 ± 0.3	1.04 ± 0.5	0.39	0.26
CXCL1	10.4 ± 2.1	6.4 ± 0.8	13.3 ± 1.8	10.23 ± 1.9	0.15	0.28
CXCL2	9.5 ± 3.0	4.5 ± 0.5	13.7 ± 2.7	12.14 ± 2.8	0.11	0.70
CXCL9	3.0 ± 0.2	2.7 ± 0.2	1.3 ± 0.1	2.12 ± 0.4	0.44	0.19
IP‐10	3.3 ± 0.2	3.3 ± 0.2	3.2 ± 0.1	3.39 ± 0.4	0.91	0.81

Results are mean ± SE of data from 6–8 mice per treatment group.

### IL‐1α accounts for effects of androgens on O_3_‐induced neutrophil recruitment but not AHR

**Figure 6:**
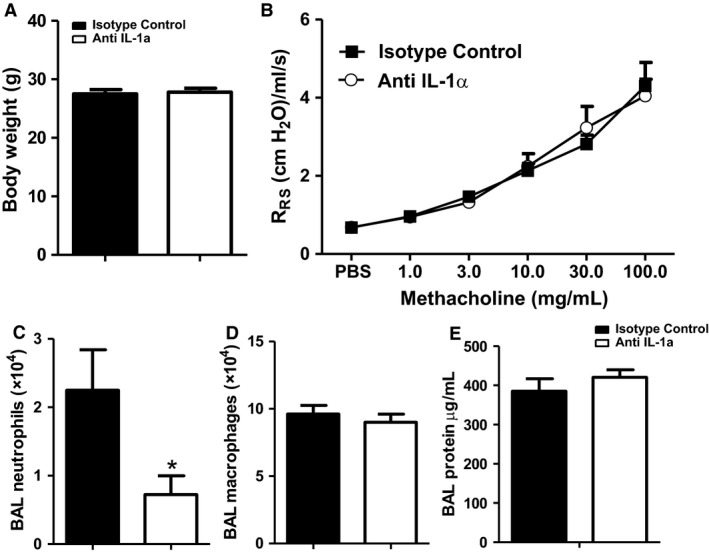
IL‐1α does not alter O_3_‐induced AHR in male mice. Gonadal intact male were treated with anti‐IL‐1α antibody or isotype control 24 h prior to exposure to O_3_ and evaluated 24 h later. Shown are: (A) body weight after treatment with isotype control or anti‐IL‐1α antibody; (B) airway responsiveness of isotype control or anti‐IL‐1α exposed to O_3_; (C) BAL neutrophils; (D) BAL macrophages; (E) BAL protein. Results are mean ± SE of data from 10 mice per treatment group. *P < 0.05 compared with isotype control.

Because IL‐1α was reduced in both castrated and flutamide‐treated mice (Fig. 5C), and because others have reported that IL‐1α contributes to O_3_‐induced AHR in female mice (Michaudel et al., 2018), we examined the effect of treatment with an IL‐1α neutralizing antibody on pulmonary responses to O_3_ in male mice. Treatment with either isotype control or anti‐IL‐1α antibody did not alter the body weight of the mice (Fig. 6A). Compared to isotype control antibody, anti‐IL‐1α had no effect on airway responsiveness in O_3_‐exposed gonadally intact male mice (Fig. 6B). However, anti‐ IL‐1α did reduce BAL neutrophils (Fig. 6C), confirming the efficacy of the anti‐IL‐1α treatment and indicating that effects of castration and flutamide on BAL neutrophils (Fig. 1C, 3D) may be related to their ability to attenuate IL‐1α (Fig. 5C). Anti‐IL‐1α had no effect on BAL macrophages or protein (Fig. 6D‐E).

### Serum corticosterone is elevated in castrated mice exposed to O_3_

**Figure 7:**
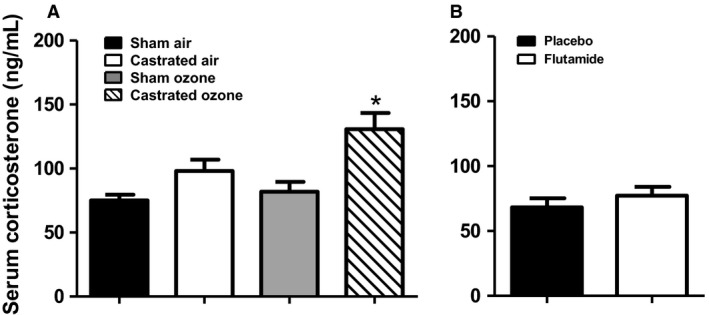
Effect of androgens on serum corticosterone. Serum corticosterone was measured in (A) sham surgery control or castrated male mice exposed to room air or O_3_ and in (B) placebo or flutamide‐treated mice exposed to O_3_. Results are mean ± SE of data from 4 air or 8 O_3_ mice per group. *P < 0.05 compared with sham O_3_‐exposed mice.

Depending on the exposure regimen, exposure to O_3_ can increase circulating concentrations of the stress hormone, corticosterone (Henriquez et al., 2017). Corticosterone release is regulated by the hypothalamic pituitary adrenal axis and is influenced by sex hormones (Seale et al., 2004): androgens inhibit stress hormone release (Handa et al., 1994). Because O_3_‐induced release of corticosterone contributes to O_3_‐induced cellular inflammation in rats (Miller et al., 2016), we considered the possibility that androgen‐induced effects on corticosterone release might contribute to the observed effects of androgens on O_3_‐induced AHR (Figs. 1, 2, 3, 4). In O_3_‐exposed mice, serum corticosterone was significantly greater in castrated versus sham‐castrated mice (Fig. 7A). In contrast, there was no effect of flutamide treatment on serum corticosterone (Fig. 7B). Since stress hormones augment rather than attenuate pulmonary responses to O_3_ in other rodents (Miller et al., 2016), and since flutamide had no effect on corticosterone but did impact responses to O_3_, it is unlikely that the elevated levels of corticosterone observed in castrated mice are responsible for the reduced responses to O_3_ observed in these mice.

### Castration and oxidative stress

**Figure 8:**
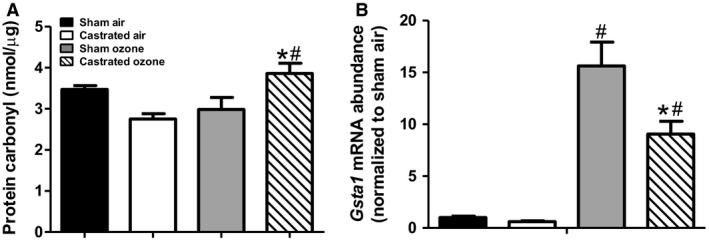
Ozone induces oxidative stress in castrated mice. Lung tissue protein carbonyl concentrations (A) and lung mRNA expression of *Gsta1* (B) were measured in sham surgery control and castrated mice exposed to room air or O_3_. Results are mean ± SE of data from 4 air or 8 O_3_ mice per group. *P < 0.05 compared to sham O_3_ exposed mice. ^#^ *P* < 0.05 compared to air exposure of same group.

In other organ systems androgens have been shown to promote oxidative stress. For example, in spontaneously hypertensive rats, castration reduces blood pressure and attenuates NADPH‐oxidase expression in the kidney resulting in reduced oxidative stress (Iliescu et al., 2007). In mice, exogenous administration of androgens increases markers of oxidative stress within the brain (Bueno et al., 2017). Women diagnosed with polycystic ovary syndrome, a hyperandrogenic disease, have less circulating glutathione and more malondialdehyde, a marker of oxidative stress than control women matched for body mass index (Sabuncu et al., 2001; Murri et al., 2013). Because O_3_ exposure promotes oxidative stress within the lungs (Sunil et al., 2012; Wiegman et al., 2014; Mathews et al., 2017), we considered the possibility that androgens act to augment O_3_‐induced AHR by increasing oxidative stress. To address this possibility, we measured protein carbonyls, a marker of O_3_‐induced oxidative stress (Cho et al., 2013), in lung tissue. Factorial ANOVA indicated a significant interaction (*P* < 0.01) between castration and O_3_ exposure on lung protein carbonyls (Fig. 8A): protein carbonyls were significantly increased by O_3_ in castrated but not sham castrated mice, and protein carbonyls were significantly greater in O_3_‐exposed castrated versus sham castrated mice. In contrast, there was no significant difference in protein carbonyls between sham and castrated air exposed mice. The absence of any O_3_‐induced increase in protein carbonyls in the sham mice is likely the result of the timing of tissue harvest, since increases in protein carbonyls peak approximately 3 h after cessation of exposure and resolve towards baseline by 24 h (Cho et al., 2013), the time point we assessed. We also measured lung mRNA expression of *Gsta1*, an antioxidant enzyme involved in glutathione conjugation and detoxification that is induced by O_3_ exposure (Mathews et al., 2017). *Gsta1* mRNA abundance was significantly greater in O_3_‐ versus air‐exposed mice, whether the mice were castrated or sham‐castrated, but the magnitude of this increase was significantly lower in the castrated than the sham‐castrated mice (Fig. 8B). Reduced expression of this antioxidant gene in the castrated mice would be expected to augment oxidative stress, as observed (Fig. 8A). The data indicate that the protective effects of castration on O_3_‐induced AHR and inflammatory cell recruitment were not the result of reduced oxidative stress in these mice.

### Androgens augment serum IL‐6

**Figure 9:**
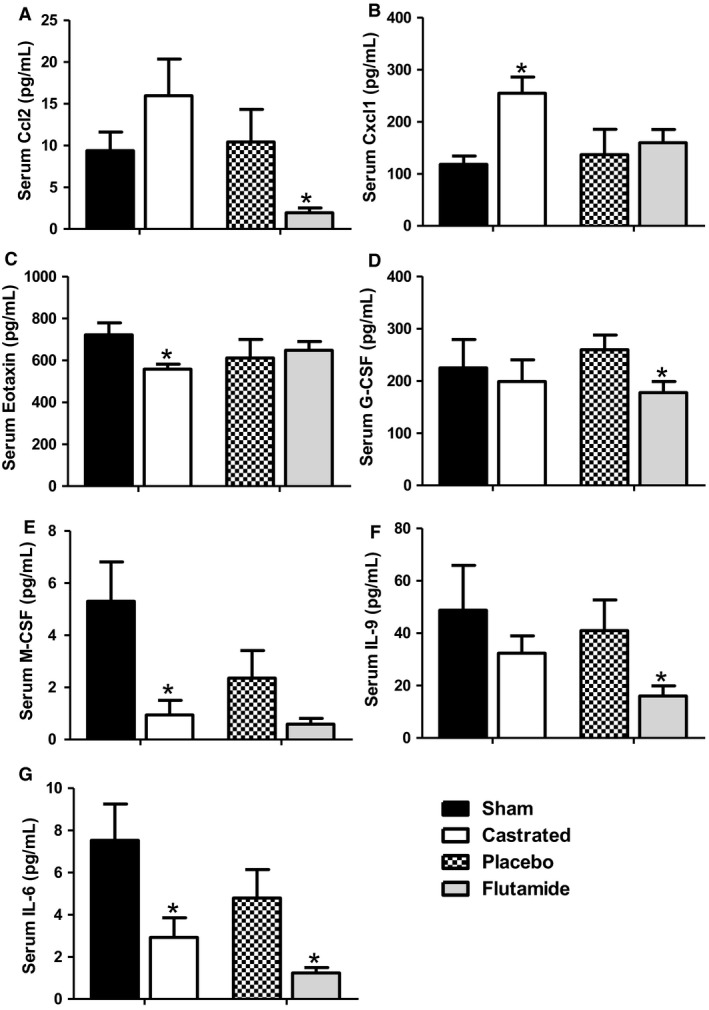
Effect of castration and flutamide on serum cytokines and chemokines in O_3_‐exposed mice. Multiplex assay was used to assess serum cytokines and chemokines in O_3_ exposed mice that underwent sham surgery or castration as well as in mice treated with placebo or flutamide. Results are mean ± SE of data from seven mice per treatment group. *P < 0.05 compared with group control (sham or placebo).

As described above, both castration and flutamide‐treatment decreased some BAL cytokines and chemokines in O_3_ exposed mice (Fig. 5). Because others have reported sex differences in systemic inflammation, which may impact airway responsiveness (Mabley et al., 2005; Williams et al., 2013), we considered the possibility that castration‐ and flutamide‐induced changes in O_3_‐induced AHR and inflammatory cell recruitment (Figs 1, 2) might be the result of effects of androgens on systemic inflammation. To address this possibility, we examined a panel of cytokines and chemokines in serum of O_3_‐exposed mice. Several serum cytokines and chemokines were significantly altered by castration or by flutamide treatment (Fig. 9). However, only IL‐6 was significantly affected by *both* treatment regimes: compared to control, both castration and flutamide significantly reduced serum IL‐6 (Fig. 9G).

## Discussion

Our data show that castration significantly decreased O_3_‐induced AHR and inflammatory cell recruitment in male mice (Figs. 1, 2) and that treatment with the androgen receptor antagonist, flutamide, had similar effects (Figs. 3, 4). The data support the hypothesis that androgens augment pulmonary responses to O_3_ and contribute to sex differences in O_3_‐induced AHR.

Both castration and the androgen receptor antagonist, flutamide, attenuated O_3_‐induced AHR. This is the first report indicating a role for androgens in augmenting O_3_‐induced AHR, though others have reported a role for androgens in augmenting innate AHR (Card et al., 2006). In addition to androgen receptor‐mediated effects on gene expression, androgens can also act on cell surface receptors including GPRC6A (Pi et al., 2010) and GPRC6A is abundantly expressed in the lungs (Kuang et al., 2005). However, the observation that flutamide, an androgen receptor antagonist, had effects on O_3_‐induced inflammation and inflammatory cell recruitment similar to those of castration suggests that effects of androgens on these responses to O_3_ are likely mediated by the androgen receptor not GPRC6A.

Neutrophilic inflammation and AHR are often disassociated (Cui et al., 2005; Starkhammar et al., 2014; Kasahara et al., 2015b). Our data further support these reports. We now report that anti‐IL‐1α treatment reduced BAL neutrophils in O_3_‐exposed mice without O_3_‐induced AHR (Fig. 6). Similarly, IL‐6 deficiency reduces BAL neutrophils but has no effect on O_3_‐induced AHR (Johnston et al., 2005b). These data suggest that the mechanisms accounting for effects of O_3_ on neutrophil recruitment versus AHR are distinct. Consequently, effects of androgens on O_3_‐induced AHR and O_3_‐induced neutrophil recruitment are discussed separately below.

The mechanistic basis for the effects of androgens on O_3_‐induced AHR remains to be established. It is unlikely that androgens augment AHR via direct effects on airway smooth muscle: others have reported that androgens relax rather than contract airway smooth muscle (Kouloumenta et al., 2006; Flores‐Soto et al., 2017). We explored the possibility that androgen‐dependent changes in IL‐1α release, systemic inflammation, corticosteroid release, or oxidative stress might be involved. Although BAL IL‐1α was reduced in both castrated and flutamide treated mice exposed to O_3_ (Fig. 5C), anti‐IL‐1α had no effect on O_3_‐induced AHR in male mice (Fig. 6B), even though it did affect O_3_‐induced neutrophil recruitment (Fig. 6C). Serum IL‐6 was reduced in both castrated and flutamide treated mice (Fig. 9G). However, while high serum IL‐6 is associated with severe asthma in humans (Peters et al., 2016) and while IL‐6 contributes to allergen‐induced AHR in mice (Lin et al., 2016), IL‐6 does not appear to play a role in O_3_‐induced AHR even though it does contribute to neutrophil recruitment (Johnston et al., 2005b).

In rats, serum corticosterone levels are elevated immediately following acute O_3_ exposure (Miller et al., 2016). We did not observe any such increases in corticosterone 24 h after exposure in sham mice, likely because of the timing of the tissue harvest. Others have shown that O_3_‐induced increases in corticosterone resolve by 24 h after cessation of exposure (Thomson et al., 2013). In contrast, corticosterone was elevated after O_3_ exposure in castrated mice (Fig. 7A). However, corticosterone has been shown to promote O_3_‐induced injury and inflammation (Miller et al., 2016), whereas the increase in serum corticosterone we observed in the castrated mice was associated with a reduction in responses to O_3_. The data indicate that androgen‐dependent effects on corticosterone release do not account for androgen‐dependent effects on O_3_‐induced AHR or inflammation.

Exposure to ozone induces the expression of antioxidant genes, such as *Gsta1*, that play a role in mitigating oxidative stress following O_3_ exposure (Cho et al., 2013). Our data demonstrate that castrated mice have more lung protein carbonyls and less mRNA expression of *Gsta1* compared with sham mice (Fig. 8). The lower expression of *Gsta1* in O_3_‐exposed castrated versus sham mice may account for the greater oxidative stress in these mice. Similarly, lower lung glutathione in O_3_‐exposed mice deficient in *Nrf2*, a transcription factor required for induction of *Gsta1*, is associated with greater oxidative stress (Cho et al., 2013). The data indicate that reduced O_3_‐induced AHR in castrated mice is not the result of reduced oxidative stress.

Androgen‐induced changes in the microbiome could also be involved in O_3_‐induced AHR. We have reported that antibiotics and germ‐free conditions both attenuate O_3_‐induced AHR in male mice (Cho et al., 2018a) and that sex differences in O_3_‐induced AHR are abolished after antibiotic treatment (Cho et al., 2018b). There are sex differences in the gut microbiome (Cho et al., 2018b)^,^(Markle et al., 2013) that only appear after puberty (Markle et al., 2013). Furthermore, castration has been shown to alter the gut microbiome (Markle et al., 2013; Org et al., 2016).

Our data indicate that in addition to effects of O_3_‐induced AHR, androgens also contribute to O_3_‐induced inflammatory cell recruitment: castration and flutamide each attenuated BAL neutrophils and/or macrophages in O_3_‐exposed mice (Figs. 1C‐D, 3D‐E). The ability of androgens to promote cellular inflammation after O_3_ exposure is novel, but is consistent with the expression of androgen receptors in both macrophages and neutrophils (Mantalaris et al., 2001). Effects of androgens on O_3_‐induced inflammatory cell recruitment may be the result of androgen‐dependent effects on IL‐1α or IL‐6. Release of IL‐1α can induce neutrophilic inflammation (Rider et al., 2011) and both castration and flutamide reduced BAL IL‐1α in O_3_‐exposed mice (Fig. 5C). Furthermore, anti‐IL‐1α treatment attenuated BAL neutrophils (Fig. 6C) in O_3_‐exposed male mice. Serum IL‐6 was also reduced by both flutamide and castration in O_3_‐exposed (Fig. 9G), and O_3_‐induced neutrophil recruitment to the lungs is attenuated in IL‐6‐deficient versus wildtype mice (Johnston et al., 2005b; Shore et al., 2009). However, we cannot rule out the possibility that the decreased inflammatory cell recruitment to the lungs of castrated or flutamide‐treated mice is the result of loss of androgen‐induced stimulation of neutrophil differentiation and production. Both male and female androgen receptor knockout mice are neutropenic (Chuang et al., 2009) and lower numbers of circulating neutrophils would be expected to reduce neutrophil recruitment to the lungs following O_3_. Indeed, LPS‐induced neutrophil recruitment to the lungs or prostate gland is also reduced in castrated versus control mice (Card et al., 2006; Scalerandi et al., 2018). We did not examine the phenotype of the neutrophils recruited to the lungs after O_3_, but others have reported that in the presence of testosterone, neutrophils recruited to the prostate gland following LPS instillation have an altered, N2, phenotype characterized by impaired bacteriocidal activity that is associated with increased IL‐10 expression (Scalerandi et al., 2018).

It is notable that the impact of castration is different for allergen‐induced versus O_3_‐induced effects on the airways. In allergen sensitized and challenged mice, allergen‐induced AHR and allergen‐induced inflammation are greater in females than in males and castration augments rather than attenuates these effects of allergen (Fuseini et al., 2018). In contrast, we and others have reported that O_3_‐induced AHR is greater in male than female mice (Cho et al., 2018a; Birukova et al., 2019) and we now report that castration attenuates rather than augments responses to O_3_ (Fig. 1). The difference likely lies in the nature of the response to these two stimuli – an adaptive immune response to allergen and an innate immune response characterized by recruitment of neutrophils and macrophages after acute O_3_ exposure. Androgens typically suppress the adaptive immune system, whereas they promote generation of neutrophils and recruitment and cytokine generation with macrophages (Lai et al., 2012).

There is increasing evidence demonstrating the importance of considering sex as a biologic variable. Our data indicate that androgens augment O_3_‐induced AHR and likely contribute to the augmented responses to O_3_ observed in male versus female mice (Cho et al., 2018a; Birukova et al., 2019). Better understanding of the mechanistic basis for this effect of androgens may ultimately lead to improved treatment of air pollutant exacerbated lung diseases such as asthma.
